# Effects of a COVID-19 Public Health Lockdown on Drinking and Health Behavior Among Persons with HIV and with Unhealthy Alcohol use in Uganda

**DOI:** 10.1007/s10461-023-04042-y

**Published:** 2023-03-31

**Authors:** Brian Beesiga, Kara Marson, Robin Fatch, Nneka I. Emenyonu, Julian Adong, Allen Kekibiina, Sarah Puryear, Sara Lodi, Michael G. McDonell, Winnie R. Muyindike, Moses R. Kamya, Judith A. Hahn, Gabriel Chamie

**Affiliations:** 1 Infectious Diseases Research Collaboration (IDRC), Kampala, Uganda; 2 Department of Medicine, University of California, San Francisco, San Francisco, CA USA; 3 Global Health Collaborative, Mbarara University of Science and Technology, Mbarara, Uganda; 4 Boston University School of Public Health, Boston, MA USA; 5 Elson S Floyd College of Medicine, Washington State University, Spokane, WA USA; 6 Mbarara Regional Referral Hospital, Mbarara, Uganda; 7 Department of Medicine, Makerere University, Kampala, Uganda; 8 Department of Epidemiology and Biostatistics, University of California, San Francisco, San Francisco, CA USA; 9 San Francisco General Hospital, University of California, San Francisco, San Francisco, CA 94110 USA

**Keywords:** Coronavirus disease-19 (COVID-19), HIV, TB, Lockdown, Unhealthy alcohol use

## Abstract

To better understand the impact of Uganda’s initial COVID-19 lockdown on alcohol use, we conducted a cross-sectional survey (August 2020-September 2021) among persons with HIV (PWH) with unhealthy alcohol use (but not receiving an alcohol intervention), enrolled in a trial of incentives to reduce alcohol use and improve isoniazid preventive therapy. We examined associations between bar-based drinking and decreased alcohol use, and decreased alcohol use and health outcomes (antiretroviral therapy [ART] access, ART adherence, missed clinic visits, psychological stress and intimate partner violence), during lockdown. Of 178 adults surveyed whose data was analyzed, (67% male, median age: 40), 82% reported bar-based drinking at trial enrollment; 76% reported decreased alcohol use during lockdown. In a multivariate analysis, bar-based drinking was not associated with greater decreases in alcohol use during lockdown compared to non-bar-based drinking (OR = 0.81, 95% CI: 0.31–2.11), adjusting for age and sex. There was a significant association between decreased alcohol use and increased stress during lockdown (adjusted β = 2.09, 95% CI: 1.07–3.11, P < 0.010), but not other health outcomes.

## Introduction

The World Health Organization declared COVID-19 a global pandemic on March 11, 2020 [1]. With this declaration, governments around the world implemented lockdown measures including the closure of schools, “non-essential” businesses and public transportation, physical distancing measures, and in some settings, strict stay-at-home orders [2, 3], in an effort to curb the spread of SARS-CoV-2. Uganda instituted a nationwide lockdown from March to June 2020 following the announcement of the first confirmed positive COVID-19 case in the country on March 21, 2020 [4, 5]. The lockdown involved the suspension of public gatherings, closure of schools and businesses including drinking establishments, discontinuation of public and private transportation, and enforcement of a national curfew [5]. However, healthcare facilities and outpatient clinics including HIV clinics continued to operate during the lockdown. Modeling suggests the lockdown was effective in slowing the spread of COVID-19 [6], but lockdown measures also impacted multiple aspects of public and private life [7, 8]. As COVID-19 vaccination rates remain low across much of sub-Saharan Africa (SSA) [9], including in Uganda, and new SARS-CoV-2 variants emerge, lockdowns remain a potential, effective public health tool to mitigate the effects of COVID-19 during times of increased transmission. Indeed, Uganda instituted a second nationwide lockdown from June to August 2021 during a COVID-19 surge driven by the Delta variant [10].

How COVID-19 lockdowns impact persons with HIV (PWH) in sub-Saharan Africa (SSA), a region with a high prevalence of HIV [11] remains incompletely understood. In Uganda, adult HIV prevalence is high at 5.8% with the prevalence in Southwestern Uganda higher than the national average at 7.3% [12]. Though PWH are at increased risk of morbidity from COVID-19 [13, 14], the effects of COVID-19 lockdowns have also included decreases in HIV testing, as well as disruptions in care delivery and treatment access for PWH [15, 16]. PWH with unhealthy alcohol use are particularly vulnerable to disruptions in care. Unhealthy alcohol use, broadly defined as any alcohol use that increases the likelihood or risk of health consequences or that has already resulted in health consequences [17], is high in SSA [18], with a prevalence of 32.2% among PWH initiating antiretroviral therapy (ART) in South Africa and Uganda [19]. Prior to the COVID-19 pandemic, PWH with unhealthy alcohol use were already at greater risk than those not drinking of non-retention in care, non-adherence to antiretroviral therapy (ART), and HIV viremia [20–22].

Furthermore, how COVID-19 lockdowns impacted alcohol use and other health outcomes among this vulnerable group of PWH in SSA is unknown. Lockdowns have the potential to operate in two distinct ways: first, increased social isolation and anxiety about the pandemic or the lockdown itself could lead to increased alcohol use [23, 24] with associated negative health impacts, including reductions in ART adherence and lack of retention in HIV care. Alternatively, closure of bars, curfews, and decreased access to alcohol as a consequence of a lockdown could reduce drinking [23, 24] with the potential for secondary positive effects on ART adherence and retention in care, as well as other health outcomes associated with decreased alcohol use, including reductions in psychological stress, and intimate partner violence (IPV) [25–27]. As alcohol use is an established risk factor for worsening mental health, decreased alcohol use could result in improvements in mental health, including reduced psychological stress. In Uganda, previous studies have linked alcohol use and IPV [25, 26], and in a recent study of IPV during the COVID-19 pandemic, adults with unhealthy alcohol use had significantly higher odds of experiencing recent physical IPV [26].

We sought to understand the impact of Uganda’s initial national COVID-19 lockdown on drinking behavior among PWH who engage in unhealthy alcohol use enrolled in an ongoing clinical trial. First, we evaluated if drinking behavior during the lockdown varied by alcohol drinking venue (i.e., bars vs. non-bar venues). Second, we evaluated if changes in drinking during the lockdown impacted health outcomes, including ART adherence, clinic visits, IPV, and psychological stress, given the known associations between unhealthy alcohol use and these outcomes [20–22, 25–27]. We hypothesized that for persons consuming alcohol at bars, the lockdown resulted in reduced drinking due to limited access from bar closures, curfews, and restrictions on mobility, whereas for those not consuming alcohol at bars, the lockdown resulted in either no change or increased alcohol use. Secondarily, we hypothesized that reduced drinking during the lockdown would be associated with greater reported positive health effects, including improvements in ART access and adherence, clinic visit attendance, psychological stress, and IPV.

## Methods

### Study Design and Setting

We conducted a cross-sectional study to evaluate the impact of the COVID-19 lockdown on drinking and health outcomes among adult PWH enrolled in the Drinkers’ Intervention to Prevent Tuberculosis (DIPT) study. DIPT (NCT03492216) is a 2 × 2 factorial randomized trial among 680 adult PWH co-infected with latent TB with unhealthy alcohol use at four HIV clinics (two urban and two rural) in Southwestern Uganda. The DIPT trial seeks to determine the effectiveness of conditional economic incentives on reductions in unhealthy drinking and/or adherence to isoniazid preventive therapy (IPT). The study design and procedures have been published elsewhere [28]. In brief, the study enrolled PWH co-infected with latent TB with unhealthy alcohol use, defined as a positive Alcohol Use Disorders Identification Test-Consumption (AUDIT-C) score (≥ 4 for men and ≥ 3 for women [28, 29]) and a positive urine ethyl glucuronide (EtG) test (an objective measure for recent unhealthy alcohol use, using a commercial dipstick with a cutoff of 300ng/mL) [30]. The AUDIT-C measures alcohol use in terms of frequency, quantity, and binge drinking (defined as consuming at least six drinks on a single occasion). Unhealthy alcohol use, as defined by a positive AUDIT-C score, has been associated with negative HIV care cascade outcomes both in the US and in East Africa in prior studies [20, 31].

### Measures

We conducted the study from August 2020 to September 2021, at participants’ first study visit post-lockdown (i.e., following the lifting of transport restrictions on public and private vehicles) using an interviewer-administered questionnaire that asked about changes (i.e., increase, decrease or no change) in alcohol use during the lockdown. The questionnaire assessed general knowledge of COVID-19, as well as changes in drinking, food insecurity, income, access to health care, intimate partner violence (IPV), and psychological stress, during the lockdown, as compared to pre-lockdown. We analyzed data from those randomized to trial arms that did not receive incentives for reductions in alcohol use, to avoid potential intervention effects on changes in alcohol use. We also included data from DIPT trial enrollment, including demographic variables, socioeconomic status (SES), drinking venue, self-reported alcohol use, and phosphatidylethanol (PEth) level – an abnormal cellular membrane phospholipid that is formed only in the presence of alcohol [32, 33]. To describe their preferred drinking venue, participants were asked where they usually drank alcohol in the last 3 months; participants were categorized as “consuming alcohol at bars” if they reported drinking at bars/drinking establishments. Participants who reported drinking alcohol at multiple locations were categorized as “consuming alcohol at bars” if the locations included bars/drinking establishments. To categorize SES, we created a household asset index based on the quality of housing, energy sources, and durable goods, using principal components analysis [34], and classified participants as high (top 20%), middle (middle 40%), and low (bottom 40%) SES.

### Primary Outcome

Our primary outcome was self-reported decrease in alcohol use during the lockdown. Self-reported decrease in alcohol use was defined as answering “yes” and “decrease” respectively to the following questions. “During the time the country was on lockdown, did you change the amount of alcohol you take?” (yes/no). Those who replied “yes” were then asked, “Did your drinking increase or decrease during the time of the lockdown?” We dichotomized this variable as decreased alcohol use versus no change/increased alcohol use during the lockdown to test our hypotheses.

### Secondary Outcomes

In secondary analyses, we evaluated self-reported health outcomes relating to ART access and adherence, missed HIV clinic visits, IPV, and psychological stress during the lockdown. *Difficulty with ART access* was defined as any difficulty obtaining medicines (i.e., a dichotomous “yes/no” outcome) during the time of the lockdown or since. *ART adherence* during the lockdown was categorized as decreased, no change, or increased ability to take ART as prescribed. Participants were asked if they *missed clinic visits* during the lockdown (yes/no). IPV was assessed using a modified version of the Conflict Tactics Scale [35], for use in Uganda [36] and to refer to the time during or after the lockdown period, as follows. *Any IPV* (yes/no) was defined by asking participants whether a sexual partner or household member had physically hurt or threatened them, or if they had been forced to have sex, or whether they had hurt or threatened a sexual partner or household member or forced anyone to have sex with them, during or after the lockdown. *Psychological stress* was assessed using a modified version of the Perceived Stress Scale-4 [37], referring to the time in the lockdown. The Perceived Stress Scale is a four-item version of the original PSS-14 with each item scored from 0 to 4, and is a valid and brief measure of perceived stress [38]. The PSS-4 scale asks about feelings and thoughts in the past one month. We modified the scale to capture the experiences during the lockdown without changing the nature of the questions. The observed alpha for the modified PSS-4 was 0.68.

### Statistical Analyses

To test our primary hypothesis, we examined associations between bar-based drinking and decreased alcohol use during the lockdown using unadjusted and adjusted logistic regression models. The main independent variable was alcohol drinking venue (any bar-based drinking versus no bar-based drinking) reported in the 3-months prior to DIPT trial baseline enrollment. The multivariable model was adjusted for participant’s sex and age, covariates chosen *a priori*. Among participants who reported decreased alcohol use, we additionally described the self-reported factors that led to the changes in drinking.

To test our secondary hypothesis, we conducted bivariate and multivariate models to evaluate associations between decreased alcohol use (yes/no) and each health outcome. For continuous outcomes (psychological stress), we used linear regression models, for binary outcomes (ART access, missed clinic visits, and any IPV), we used logistic regression models, and for ordered outcomes (ART adherence), we used ordered logistic regression models. We also assessed the association between changes in income during the lockdown and decreased alcohol use, as well as each health outcome. The final multivariable health outcome models assessing the associations between decreased alcohol use and the health outcomes of interest were adjusted for age, sex, and changes in income.

This study was approved by the Institutional Review Boards of the University of California San Francisco, Makerere University School of Medicine Research and Ethics Committee, Mbarara University of Science and Technology Research Ethics Committee, and the Uganda National Council for Science and Technology. All participants provided written informed consent, in either English or Runyankole, to participate in the survey.

## Results

A total of 180 of the 339 participants that did not receive incentives for alcohol reduction in the DIPT trial participated in the COVID-19 survey between August 2020 and September 2021. Two of these participants were missing data on alcohol use in the COVID-19 survey and were excluded here, leaving 178 participants for inclusion in this analysis. Overall, 120 (67%) were male, with a median age of 40 years (interquartile range [IQR 32–46]). At parent trial enrollment, median AUDIT-C score in this sample of survey participants was 6 [IQR 4–8], and median PEth level was 333 ng/mL [IQR 99–740]. Overall, 146 (82%) participants reported typically drinking at bars, of whom 28 (19%) also reported drinking at home, and 19 (13%) also reported drinking at parties. During the COVID-19 lockdown, 164 (93%) reported a decrease in their income and 74 (42%) reported difficulty in obtaining food. However, 144 (81%) reported no difficulty in accessing ART, 170 (96%) reported no change in ART adherence, and 160 (90%) reported no missed clinic visits. The median psychological stress score was 13 [IQR 9–14] with a higher score indicating a higher level of stress (up to a maximum score of 16), and 19 (10%) of the participants reported IPV. Overall, only 4 (2%) reported ever having had a positive COVID-19 diagnosis at the time of the survey (Table I).

**Table I:** Characteristics of participants in a cross-sectional survey examining the effects of Uganda’s initial national COVID-19 lockdown on drinking and health behavior among persons with HIV and unhealthy alcohol use in Uganda.

Participant characteristics (n = 178).	
	N (%)
Sex Female Male	58 (32.6%) 120 (67.4%)
Age (median [IQR])	39.5 [32–46]
Household asset index Bottom Middle Top	82 (46.1%) 63 (35.4%) 33 (18.5%)
COVID-19 diagnosis No Yes	174 (97.8%) 4 (2.3%)
**Alcohol use at study enrollment**	
AUDIT-C (median [IQR])	6 [4–8]
PEth ng/mL (median [IQR])	333 [99–740]
AUDIT-C > = 6 and/or PEth > = 200 ng/mL No Yes	40 (22.5%) 138 (77.5%)
Consumes alcohol at bars? No Yes	32 (18.0%) 146 (82.0%)
**During COVID-19 lockdown vs. pre-lockdown**	
Alcohol use decreased No Yes	42 (23.6%) 136 (76.4%)
Income decreased No Yes	13 (7.3%) 164 (92.7%)
Difficulty obtaining food No Yes	104 (58.4%) 74 (41.6%)
Difficulty with ART access No Yes	144 (82.3%) 31 (17.7%)
ART adherence Decreased No change Increased	5 (2.8%) 170 (96.1%) 2 (1.1%)
Missed clinic visits No Yes Don’t know	160 (89.9%) 16 (9.0%) 2 (1.1%)
Intimate Partner Violence (any) No Yes	159 (89.3%) 19 (10.7%)
Mental health (Perceived Stress Scale-4; median [IQR])	13 [9–14]

### Alcohol use During the Lockdown

Of 178 survey participants, 136 (76%) reported a decrease in alcohol use during the COVID-19 lockdown. The reasons participants cited for decreased drinking during the lockdown (not mutually exclusive) primarily related to challenges to accessing alcohol: 65 (48%) reported decreased availability of alcohol, 47 (35%) reported worry/stress related to stay-at-home restrictions (including fear of enforcement by the police/authorities), and 41 (30%) reported changes in the ability to be out at night (due to a lockdown curfew). The other major reason that participants cited for decreased drinking was fear of getting COVID-19 (52 [38%]).

No significant associations were observed between decreased drinking versus unchanged/increased drinking and typical drinking venue (i.e., bar-based or non-bar-based), age and sex in the unadjusted analyses. In the multivariate analysis, bar-based drinking was not significantly associated with a decrease in alcohol use during the lockdown (aOR 0.81, 95% CI: 0.31–2.11, p = 0.660), after adjusting for age and sex (Table II).

**Table II:** Bivariate and multivariable associations with decreased alcohol use among persons with HIV and unhealthy alcohol use during Uganda’s initial national COVID-19 lockdown.

Participant characteristics (N = 178)	Alcohol use during lockdown			
	Same/increased (n = 42)	Decreased (n = 136)		
	N (%) or median [IQR^a^]	N (%) or median [IQR]	Unadjusted Odds Ratio (OR) (95% CI^b^)	Adjusted OR (95% CI)
Consumes alcohol at bars				
No	7 (21.9)	25 (78.1)	1.00	1.00
Yes	35 (24.0)	111 (76.0)	0.89 (0.35, 2.23)	0.81 (0.31, 2.11)
Sex				
Female	14 (24.1)	44 (75.9)	0.96 (0.46, 2.00)	0.84 (0.38, 1.85)
Male	28 (23.3)	92 (76.7)	1.00	1.00
Age	40.5 [32–50]	39 [32-45.5]	0.99 (0.95, 1.02)	0.98 (0.95, 1.02)

^**a**^ IQR: Interquartile range.

^b^ CI: Confidence interval.

### Effects of Decreased Alcohol use and Health Outcomes

Although 136 participants (76%) self-reported a decrease in alcohol use during the lockdown, we did not find any significant association between reduced drinking and health outcomes including ART access (OR 2.25, 95% CI: 0.74–6.87, p = 0.150), ART adherence (OR 0.54 95% CI: 0.08–3.75, p = 0.540), missed clinic visits (OR 1.35 95% CI: 0.37–4.99, p = 0.650) or IPV (OR 2.86 95% CI: 0.63–12.91, p = 0.150) in the unadjusted analyses. However, decreased alcohol use was significantly associated with greater stress on the Perceived Stress Scale (PSS-4) (median of 14 among those reporting decreased alcohol use vs. 8.5 among those reporting no decrease in alcohol use, β = 2.14, 95% CI: 1.08–3.20, p < 0.010).

In multivariate analyses, a significant association remained between self-reported decrease in alcohol use during the lockdown and increased stress (adjusted β = 2.09, 95% CI: 1.07–3.11, P < 0.010), after adjusting for age, sex, and income. Decreased alcohol use was not associated with the other health outcomes we explored: ART access (aOR 2.17 95% CI: 0.70–6.67, p = 0.180), ART adherence (aOR 0.51, 95% CI: 0.07–3.58, p = 0.500), missed clinic visits (aOR 1.16, 95% CI: 0.30–4.39, p = 0.830) and IPV (aOR 2.97, 95% CI: 0.64–13.88, p = 0.170). Male participants had significantly higher odds of self-reported ART adherence compared to females after adjusting for age, income, and self-reported alcohol use (aOR 10.46, 95% CI: 1.15–95.51, p = 0.040) (Table III).

**Table III:** Multivariate associations between decreased alcohol use and health outcomes among persons with HIV and unhealthy alcohol use during Uganda’s initial national COVID-19 lockdown.

Participant characteristics	Difficulty with ART access (n = 174)*		Decreases in ART adherence (n = 176)**		Missed clinic visits (n = 176) ^α^		Any IPV (n = 177) ^x^		Perceived stress scale (n = 177) ^x^	
	Adjusted OR (95% CI)	p-value	Adjusted OR (95% CI)	p-value	Adjusted OR (95% CI)	p-value	Adjusted OR (95% CI)	p-value	adjusted β (95% CI)	p-value
Alcohol use decreased		0.180		0.500		0.830		0.170		< 0.010
No	1.00		1.00		1.00		1.00		ref	
Yes	2.17 (0.70, 6.67)		0.51 (0.07, 3.58)		1.16 (0.30, 4.39)		2.97 (0.64, 13.88)		2.09 (1.07, 3.11)	
Sex		0.380		0.040		0.090		0.290		0.060
Female	1.00		1.00		1.00		1.00		ref	
Male	1.50 (0.61, 3.72)		10.46 (1.15, 95.51)		3.26 (0.82,12.89)		0.58 (0.21, 1.60)		-0.81 (-1.85, 0.04)	
Age (per 1 year)	1.00 (0.96, 1.04)	0.930	1.00 (0.92, 1.08)	0.980	0.94 (0.89, 1.00)	0.070	0.96 (0.90, 1.01)	0.110	-0.02 (-0.07, 0.02)	0.330
Income		0.430		0.900				0.750		0.170
Similar/higher than usual	1.00		1.00		-		1.00		ref	
Lower than usual	2.34 (0.28, 19.72)		0.80 (0.03, 25.28)		-		0.70 (0.08, 6.42)		1.20 (-0.50, 2.91)	

**Abbreviations**: ART: Antiretroviral therapy; IPV: Intimate Partner Violence; OR: Odds Ratio; CI: Confidence Interval.

*n = 3 missing ART access data; n = 1 missing income data

**n = 1 missing ART adherence data; n = 1 missing income data

^α^n = 2 missing missed visits data

^x^ n = 1 missing income data

## Discussion

In a cross-sectional survey of PWH with unhealthy alcohol use in Uganda, we found that over three-quarters of survey respondents reported decreased alcohol use during Uganda’s initial (2020) national COVID-19 lockdown. The reported decrease in alcohol use was driven primarily by structural challenges to accessing alcohol because of the lockdown, and concerns about acquiring COVID-19; reported loss of income during the lockdown may have also contributed. During the lockdown, the Ugandan government closed drinking venues and instituted curfews [5], both of which likely decreased the availability of alcohol and reduced the ability of people to drink within social contexts. Despite these lockdown measures, we did not observe significant differences in reduced alcohol use among PWH who reported bar-based versus no bar-based drinking, suggesting that these widespread structural barriers to alcohol use affected persons with unhealthy alcohol use independent of drinking venue type. Furthermore, despite potential health benefits that we hypothesized might follow from decreased alcohol use in PWH, including improved ART adherence and decreased IPV, participants did not report significant improvements in measured health outcomes. This finding may be a combined result of successful efforts to maintain HIV care during the lockdown (as reflected by the high proportion of participants reporting no challenges accessing ART), as well as the reported negative effects of the pandemic and lockdown on income and psychological stress measures that affected most participants. Overall, our findings provide a mixed picture of the COVID-19 pandemic and lockdown effects on this vulnerable population of PWH with unhealthy alcohol use, with evidence of well-maintained access to HIV clinical services and of decreased alcohol use, but also negative impacts on income and psychological stress.

Literature on the effect of COVID-19 lockdowns and other mitigation measures on alcohol use in Sub-Saharan Africa has been limited to date. In a recent systematic review by Sohi et al., there was substantial heterogeneity in changes in alcohol use in response to the COVID-19 pandemic; the authors noted a lack of studies from low- and middle-income countries [39]. Similarly, a recent meta-analysis of studies evaluating changes in alcohol use noted substantial heterogeneity across 58 countries that were moderated by per capita gross domestic product and country [40]. We sought to address gaps in understanding pandemic effects on alcohol use in an East African context by contributing findings from a particularly high-risk group of PWH – those with unhealthy alcohol use – in rural Uganda. Early in the pandemic, Rehm et al. hypothesized that two main scenarios of alcohol use might follow from the COVID-19 pandemic [23]. First, alcohol use might increase in response to the pandemic, because of stress and anxiety induced by a new pandemic. Alternatively, alcohol use might decline due to decreased access to alcohol and decreased income. The authors postulated that the second scenario might predominate early in the pandemic in the context of lockdowns, while the first scenario would predominate in the mid-to-long term as lockdowns lifted [23]. Our findings are consistent with this second scenario during a national COVID-19 lockdown in Uganda, regardless of drinking venue type. Our findings are also consistent with a study in central Uganda that explored the impact of COVID-19 on alcohol use among women aged 13–79 years between June to August 2020 irrespective of HIV status, in which 60% of respondents reported a decrease in alcohol use during the pandemic [26]. Further data are needed to understand how alcohol use has changed since the lockdown in Uganda was lifted.

Decreased drinking during the lockdown did not differ by bar-based vs. non-bar-based drinking in our study population. There are several possible explanations for this finding. First, the proportion of those who did not typically drink at a bar-based venue was low and may have reduced our ability to detect a difference in drinking by venue type. Second, in Uganda, people who drink at home often get alcohol from local bars, which were also closed during the national lockdown. Third, during the lockdown, many people lost their jobs owing to the closure of businesses, resulting in income loss [8]; indeed, in our sample, 93% of participants reported a decrease in their income during the lockdown. This likely resulted in prioritizing available disposable income for other basic needs in a setting where 41% of participants reported difficulties obtaining food. Fourth, our survey measured changes in drinking during the lockdown, compared to non-lockdown drinking, but did not quantify the amount of change in drinking. Thus, if the amount of alcohol use by which persons decreased their drinking was greater (or lesser) among those reporting bar-based vs. non-bar-based drinking, we may not have detected relative differences. Lastly, changes in alcohol use were based on self-report and subject to social desirability bias, which has been reported in several studies in this population [19, 41–43].

Our study found a significant association between decreased drinking and increased stress, but not other measured health outcomes. Despite potential health gains from reductions in alcohol use, there were multiple competing influences on the health outcomes we measured. Several factors likely contributed to the increase in psychological stress we observed, despite decreased alcohol use, including major disruptions in people’s public and private life, fear of a new pandemic, and loss of income. Any mental health benefits from decreased alcohol use were likely far outweighed by these stressors. Furthermore, alcohol may have been a maladaptive mechanism for coping with stress among some persons with unhealthy alcohol use in our study. Thus, a reduction in drinking due to decreased access to alcohol could have removed this “coping” strategy in response to stressors. In addition, our other health outcome measures focused on HIV healthcare access, including ART access and missed clinic visits. Early in the pandemic, there were several health systems adaptations in Uganda (by the government and implementing partners) to ensure the continuity of HIV services, including increases in community client-led ART delivery and multi-month dispensing of ART [44, 45] that may have maintained access to clinical care and ART access, and offset measurable effects of changes in drinking behavior on these outcomes in our study population. In contrast, several studies have observed health effects associated with decreased access to alcohol during COVID-19 lockdowns in SSA. In South Africa, a country in which alcohol sales were prohibited during an initial COVID-19 lockdown, along with unnecessary travel and gathering, there were decreases in emergency room visits for trauma-related injuries and unnatural deaths during the lockdown [46–48]. A study in central Uganda, which enrolled women, observed increases in reported IPV during the pandemic and associated lockdowns [26]. Moreover, the experience of physical IPV was significantly associated with unhealthy alcohol use. Whereas the relationship between alcohol use and IPV has been previously established [49], we did not find this association in our study. It is possible that any effect of reduced alcohol use on IPV was counteracted by the increased psychological stress during the lockdown that we observed in our study population, as increased psychological stress, including COVID-19 related stress, has been associated with increased IPV in previous studies [50, 51].

How changes reported in drinking behavior in the short term during the COVID-19 pandemic will impact health outcomes in the long term remains unclear. In the face of severe communicable disease outbreaks in Uganda, including emerging COVID-19 variants, lockdowns remain a public health tool used to contain epidemics and reduce transmission. For example, localized, district-wide lockdowns were imposed in several districts in Uganda in September and October 2022, to contain an Ebola virus outbreak [52]. As such, understanding the broader health effects of lockdowns and developing strategies to mitigate potential negative effects during periods of lockdown, remain important even as prior COVID-19 lockdowns have been lifted.

Our study has limitations. First, our study was cross-sectional, limiting our ability to clearly understand potential causal relationships between decreased alcohol use and the health outcomes measures. Second, as noted above, our outcomes were based on self-report and since the questionnaire was interviewer-administered, it may have been subject to social desirability bias. However, the study was conducted among participants who already reported unhealthy alcohol use (as an eligibility criterion for the parent trial), and as such, they may have been more likely to openly report drinking behavior. Third, our study recruited participants enrolled in a randomized control trial of incentives for alcohol reduction. To avoid trial intervention effects, our study excluded trial participants receiving the alcohol reduction intervention, though it remains possible that participants in the present study still possessed some motivation to modify their alcohol use by virtue of participating in the parent trial, which may reduce the generalizability of our findings to all PWH with hazardous alcohol use. Lastly, our survey measured changes in drinking during the lockdown, compared to non-lockdown drinking, but did not quantify the amount of change in drinking. However, we believe the changes reported were likely large since subtle changes tend to be unreported because of recall and social desirability biases. Our study also has strengths. We describe how the COVID-19 lockdown affected PWH co-infected with latent TB who engage in unhealthy alcohol use: a vulnerable group at particularly high risk of adverse outcomes. Additionally, we describe changes in drinking, factors that affected drinking behavior, and a range of non-COVID-19 health outcomes during a COVID-19 lockdown in a low-income setting – topics about which we continue to learn as the pandemic and mitigation measures continue to evolve.

In conclusion, during Uganda’s initial national COVID-19 lockdown, our study population of PWH with unhealthy alcohol use reported reduced alcohol use irrespective of drinking venue, and decreased alcohol use primarily resulted from lockdown-imposed barriers to alcohol procurement. Despite potential health gains from decreased alcohol use, we did not observe associations between decreased drinking and reported health outcomes, apart from increases in stress, likely due to multiple competing influences on measured health outcomes during the early stages of the COVID-19 pandemic and national lockdown in Uganda. Our findings highlight the need for stress management interventions during periods of lockdowns in order to maximize the benefits of reduced alcohol use among vulnerable groups. Stress management interventions such as stress reappraisal might be effective during pandemics at mitigating both short-term and long-term adverse health outcomes [53] and could enhance the benefits of reduced alcohol use during lockdowns.

## Data Availability

A complete de-identified dataset sufficient to reproduce the study findings will be made available upon request to the corresponding author, following approval of a concept sheet summarizing the analyses to be done.
